# An Online Health Community for Aneurysmal Subarachnoid Hemorrhage Patients: A Pilot Study

**DOI:** 10.2196/resprot.3736

**Published:** 2014-11-13

**Authors:** Hieronymus Boogaarts, Willemijn van Nuenen-Platvoet, Leonie van den Abbeele, Harriette Petersen, Irena Draskovic, Joost de Vries, Gert Westert, J Andre Grotenhuis, Ronald Bartels

**Affiliations:** ^1^Neurovascular InstituteDepartment of NeurosurgeryRadboudumcNijmegenNetherlands; ^2^Scientific Institute for Quality of Healthcare (IQ healthcare)Scientific Institute for Quality of Healthcare (IQ healthcare)RadboudumcNijmegenNetherlands

**Keywords:** subarachnoid hemorrhage, online community, quality of care

## Abstract

**Background:**

Aneurysmal subarachnoid hemorrhage (aSAH) is a condition affecting relatively young patients and has high rates of morbidity and mortality. Online health communities have emerged to fill the void for patient advocacy and information, allowing individuals with shared experiences and chronic disorders to connect.

**Objective:**

We have developed an online health community for aSAH patients, and this pilot study was conducted to evaluate it from a patient’s perspective.

**Methods:**

We implemented an online, members-only, health community (MijnSAB, translation: MySAH) in addition to the usual aSAH care at Radboudumc, Nijmegen, the Netherlands. A questionnaire that was sent to consecutive aSAH patients was used to evaluate the usability and utility of MySAH. Answers were provided using a 5-point Likert scale. There was also one open-ended question asking about what was missing from the MySAH tool.

**Results:**

In total, 66 consecutive patients with aneurysmal subarachnoid hemorrhage were informed about the online health community. Of 64 potential MySAH users, 26 patients gained access to MySAH, 20 of whom were willing to participate in the evaluation. Those who used the community were younger (*P*=.03) and in a better condition at discharge (*P*=.03). The patients were positive about MySAH’s contribution to the quality of their care, but not to their quality of life. Most patients (18/20, 90%) reported that they would recommend the community to others in their position. Open suggestions on how to improve the tool included more frequent blogs, including by a rehabilitation specialist.

**Conclusions:**

This pilot study showed that the online health community, MySAH, has a beneficial effect on the aftercare of patients suffering from aSAH because it gives easy access to relevant information provided by peers or caregivers. Due to the variable clinical outcomes after aSAH, the tool will mainly be useful for a select group of patients (with a better clinical outcome).

## Introduction

The incidence of aneurysmal subarachnoid hemorrhage (aSAH) is approximately nine cases per 100,000 [1]. The condition affects relatively young patients, with an average age at first onset of 55 years, and has significant rates of morbidity and mortality [1]. Health-related quality of life is also significantly reduced compared to the normal population [2-4]. Online health communities are increasingly being used to assist these patients and can be a valuable addition to the standard clinical and outpatient care [5-7]. Such communities give patients the opportunity to communicate with professionals and peers and to learn more about their disease and future expectations [8].

We have developed an online health community for aSAH-patients, and this pilot study was conducted to evaluate it from a patient’s perspective.

## Methods

### Implementation

**Figure 1:**
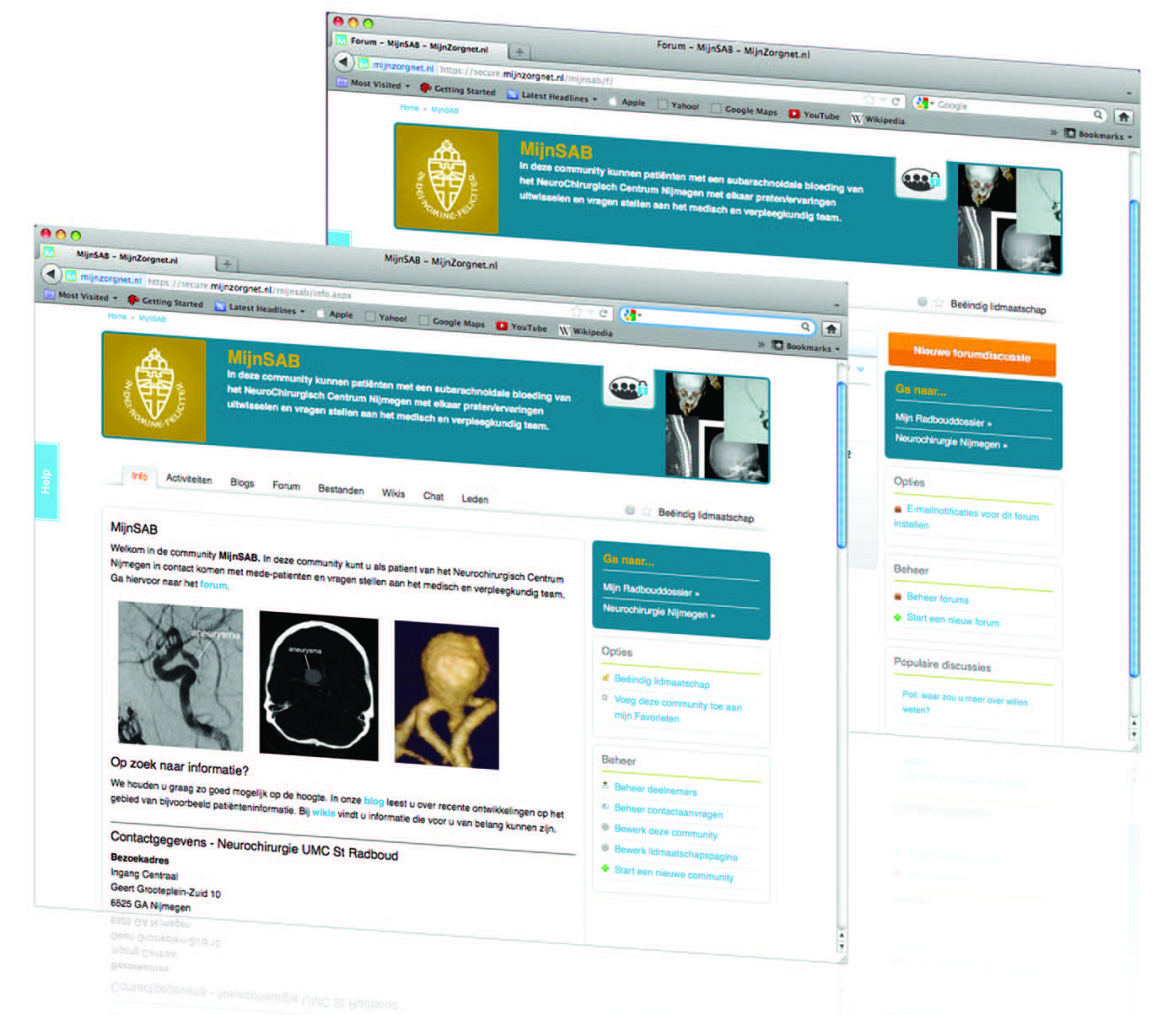
Poster of access page.

We implemented an online, members-only health community (MijnSAB, translation: MySAH) as an addition to the usual aSAH care provided at Radboudumc. The tool has three main functionalities, which are in line with those described for other online communities [5]. First, information on relevant news can be provided in blogs. Second, the resource is an interactive forum whereby patients can contact others with the disease or put questions to the medical team. Third, general information concerning several aspects of the disease is provided. An example of the access page in the form of a poster used for promotion purposes among patients is shown in Figure 1. Examples of the translated content (“Can I?”) are set out in Multimedia Appendix 1.

The community tool has been used by Radboudumc to improve patient-centered care in a number of different medical specialties, with ParkinsonNet being an example [7]. The technical maintenance costs are € 5000 per annum.

In our MySAH community, patients logged in to the site using a personal digital identification code. Attention was drawn to new messages by pop-ups in a patient’s mailbox. Two physician assistants and a nurse practitioner were responsible for daily communication with the community. Weekly checks on the Web-blogs were made by two neurosurgeons. General questions without the need for the intervention of a neurosurgeon were answered by the physician assistants or nurse practitioner. If the answers to questions required more specialist knowledge, responses were provided by one of the two neurosurgeons.

We used a questionnaire, which was sent to consecutive aSAH patients, to evaluate the usability and utility of MySAH. If patients were unable to complete the questionnaire, their caregivers were asked to do it for them. Those who did not return a completed questionnaire within 3 weeks were contacted by telephone and asked why. An eventual telephone evaluation was conducted by a physician assistant who was not involved in the treatment of the patients.

All of the aSAH patients referred to Radboudumc between November 1, 2012, and September 30, 2013, were candidates for participation, and all survivors were invited to take part in the research. The demographics of all of the referred patients were registered, as were the modified Rankin scale (mRS) at discharge and the type of post-hospital care (home, rehabilitation, or nursing home). The mRS is frequently used in aSAH patients to score outcomes and is an ordinal scale varying from 0 to 6 (0=No symptoms; 1=No significant disability. Able to carry out all usual activities, despite some symptoms; 2=Slight disability. Able to look after own affairs without assistance but unable to carry out all previous activities; 3=Moderate disability. Requires some help but able to walk unassisted; 4=Moderately severe disability. Unable to attend to own bodily needs without assistance and unable to walk unassisted; 5=Severe disability. Requires constant nursing care and attention, bedridden, and incontinent; and 6=Dead). The mRS scores have been dichotomized as ≤3 and ≥4 because it was assumed that patients with a score of more than 3 would use the Internet less often. Approval for the study was obtained from the local medical ethics committee (CMO Arnhem-Nijmegen).

### Questionnaire

We developed a questionnaire that had two parts. The first of these contained general questions on perceived care, while the second asked questions on the usability and usefulness of the MySAH community. The questions were adapted from a previously published patient agreement questionnaire containing usability- related and usefulness-related statements and were expanded for use with MySAH [9]. Answers were given using a 5-point Likert scale, the data were summarized by a median, and for the analysis, the results were collapsed in two categories, with the neutral score counted on the negative side (agree/disagree). The results are presented graphically with median and interquartile ranges [10]. There was one open-ended question about what was missing from the MySAH tool. If possible, the responses were classified according to the three components of the community and with respect to suggested technical alterations.

## Results

### Included Patients

**Figure 2:**
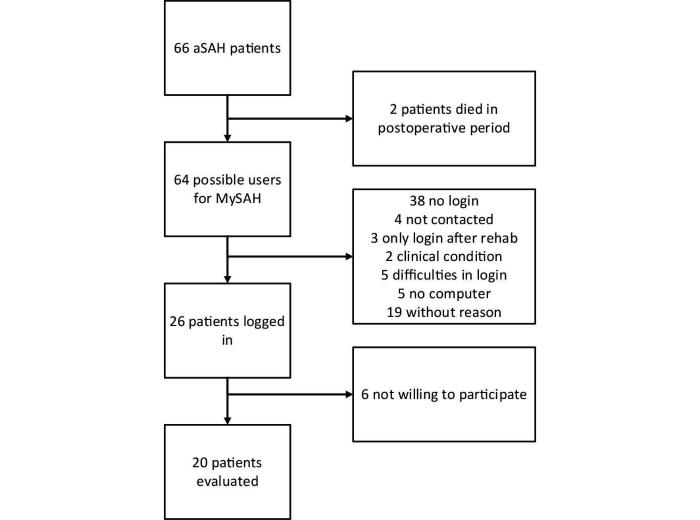
Flow chart of patients included in study.

In total, 66 patients with aneurysmal subarachnoid hemorrhage were informed about the online health community. Two patients died in the post-discharge period. Of the 64 remaining potential MySAH users, 38 did not log in, 4 could not be contacted in the post-operative period, 3 were willing to log in after a rehabilitation period, 2 did not log in because of their clinical condition, 5 had technical difficulties logging in, 5 did not have a computer, and 19 did not provide a reason for their non-participation. Finally, 26 patients did gain access to MySAH, 20 of whom were willing to participate in the study (Figure 2). The demographics of the patients, stratified by their participation, are shown in Table 1.

The participants who evaluated MySAH were not significantly different in terms of their gender or discharge location (*P*=.33). However, those who did participate were younger (*P*=.03) and were in a better clinical condition (mRS) at discharge (*P*=.03).

**Table 1:** Patient characteristics.

Characteristics		Participant	Non-participant	Total	*P* value for calculated difference between groups^a^
Number of patients, n (%)		20 (30)	46 (70)	66 (100)	Not applicable
Male/female, n/n		6/14	17/29	23/43	.78^b^
Age in years, median (SD)		48.5 (11.7)	56.0 (11.9)	54.0 (12.2)	.03^c^
**mRS at discharge, n**					.03^b^
	≤3	19	31	50	
	≥4	1	15	16	
**Discharge, n**					.33^b^
	Home	14	22	36	
	Rehabilitation	5	18	23	
	Nursing home	1	6	7	

^a^Values are considered to be significant if *P*<.05.

^b^Fisher’s Exact test (2-sided).

^c^Mann-Whitney U test (2-tailed).

### Patient Satisfaction and the Use and Usability of MySAH

The MySAH community was used for a mean period of 7.2 months, mainly bi-monthly (9/20, 45%) or monthly (7/20, 35%). A minority used the tool weekly (3/20, 15%) or daily (1/20, 5%). In most cases (16/20, 80%), the patient was the main user of MySAH, while the other responders were proxies. No specific part of the MySAH community was used preferentially by either the patients (wiki: 4/20, 20%; forum: 10/20, 50%; blogs: 2/20, 10%; not answered: 2/20, 10%) or their proxies (wiki: 3/20, 15%; forum: 2/20, 10%; blogs: 2/20, 10%; and not answered: 12/20, 65%).

**Figure 3:**
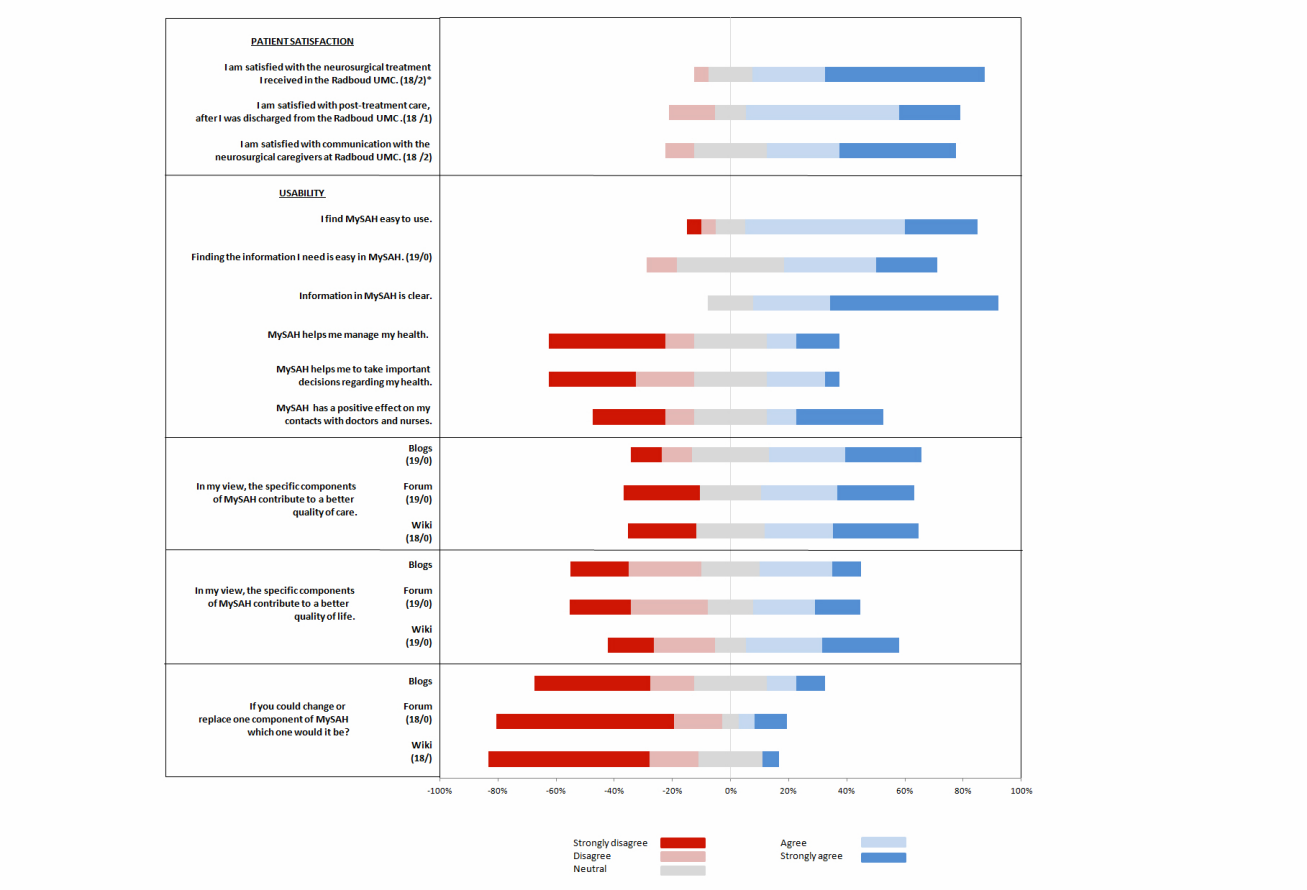
Patient satisfaction and usability (results of the questionnaire are depicted as a set of diverging stacked bar charts. Each stacked bar is 100% wide and partitioned by the percent of that group who have selected the agreement level indicated in the legend below the body of the plot. The legend is ordered by the values of the labels. Asterisk=answer by number of patients/caregivers).

The questionnaires were mainly completed by the patients and in a minority of cases by their caregivers. Patient satisfaction with treatment, post-treatment care, and communication with caregivers was generally rated positively (see Figure 3).

The information was easy to use (4.0) and find (4.0) and was also clear (5.0). However, it was not beneficial for managing health, making important decisions regarding health (2.5), or making contact with caregivers (3.0). Patients were positive about MySAH’s contribution to the quality of their care, but not to their quality of life. No specific component (blog, forum, or wiki) was preferentially rated, nor did the patients discard one aspect in particular. Most patients (18/20, 90%) would recommend the community to others in their position.

### Open Remarks

In total, 16 patients made 21 suggestions for future improvements to the community (Table 2). These responses were classified according to the three components of the community and with respect to suggested technical alterations. More frequent blogs, including by a rehabilitation specialist, was one suggestion. The forum could apparently also benefit from use by a larger number of patients overall and by patients with more positive disease experiences. The wiki section should contain more information about aftercare, psychological consequences when at home, pregnancy after aSAH, current news, and more general factors. Other suggestions were related to login and layout and navigation on the site.

**Table 2:** Items for improvement^a^.

Item	Suggestions
Blogs	More blogs (1), in combination with a rehabilitation specialist (2).
Forum	More patient contact (1), also positive experiences (1).
Wiki / information	More information on aftercare (2), psychological consequences at home (3), pregnancy after aSAH (1), current news (2), and general information (3).
Technical	Login (1), navigation (1), layout (3).

^a^Numbers in parentheses = number of patients who suggested this improvement.

## Discussion

### Principal Findings

The conceptual framework of the online community, MySAH, is to improve patient care and obtain better clinical outcomes through optimizing engagement of the patient with the treatment. This is accomplished by an exchange of information between patient and caregiver and vice versa. Such a concept is comparable with sociological studies in other fields [11].

This online health community has promising features. Although the number of responses to the questionnaire was not high (30% responders), the majority graded the items concerning usability as good. The response rate is probably related to the clinical outcome after aSAH; the patients using the community were generally in better health, which means that it may not be valuable for those in a worse condition. The users of the community were also younger, which is generally the case with health-related Internet use [12].

At our center, the treatment of aSAH patients is carried out by a subspecialist team working in a multidisciplinary setting. This team consists of neurologists, neurosurgeons, neuroradiologists, neurorehabiliation specialists, neurointensivists, and a dedicated nursing team using a protocolized aftercare program. This probably contributes to patient satisfaction with treatment, post-treatment care, and communication with caregivers. As important decisions are already taken within this framework, it is likely that no additional benefit of the online health community was identified with respect to managing health, making decisions regarding health, or making contact with caregivers. Moreover, a recent study investigating the use of an online forum identified a participant’s motivation to seek out information as one of the factors related to participation in an online community [13].

However, the patients were positive about MySAH’s contribution to their quality of care. Indeed, with the increasing centralization of subspecialized care, this online health community can provide additional, easy access (after-) care at a distance without the need to travel [14]. This was also emphasized in the open suggestions made by the patients concerning how to improve communication with the specialists (rehabilitation specialist) involved with health care after aSAH, and could be valuable in a future online health community. Such a tool would enable answers to be provided quickly on apparently less important, but for the patient at their stage of rehabilitation, very relevant issues (eg, washing hair, biking, sex). MySAH might also serve as a tool for self-management whereby patients are helped to gain control over their lives [7]. Additionally by implementing and evaluating this online community, patient engagement has led to advancements in the aftercare, especially by improved and tailored information. A lesson learned: for future caregivers starting a community, careful selection of the possible participants and their needs is paramount.

As indicated in other publications, household Internet access in the Netherlands is about 92% and should therefore be a minor limitation with respect to access to an online health tool [7,15]. Indeed, this is in line with our data in which only five of 66 patients (7.6%) did not have a computer. However, in some other states in the European Union, Internet access is less, down to 45%, and might therefore be a restricting factor in the success of such an online community [7,15].

Health-related quality of life is significantly reduced in patients with aneurysmal subarachnoid hemorrhage [2,3,16]. Important factors associated with this are physical health issues, depression, cognitive impairment, anxiety, and fatigue [2,3,17-19], and standard aftercare and rehabilitation focuses on these problems. These impairments may, however, have been barriers to the use of the community by those who might potentially benefit from it. Those who did participate evaluated the tool neutrally, regarding their quality of life as neutral.

### Limitations

This research has several limitations. First, the number of patients evaluated was only 20, as some of those approached were unwilling or unable to use the MySAH tool. However, within this pilot study, this outcome highlighted the limitations of the community in this patient category. Moreover, for evaluation purposes, having 20 participants is considered to be adequate [9]. Second, usability was self-reported, although from a quality of life perspective the use of subjective experiences is important [17]. Third, the online health community was used as an additional aftercare program and might have experienced some redundancy.

Future studies should assess the value of this online health community when fully integrated in, and as an adjunct to, face-to-face interactions. This could tailor aftercare to the wishes of the patient, enabling more patient-centered care. In our view, it should be emphasized that face-to-face contact continues to be essential in order to precisely determine outcomes and identify possible neurological deficits. Moreover, we envisage a broader use for the MySAH community in other centers involved in aSAH care in the Netherlands. Certainly, the general sections of the site could productively be used by patients from other centers, and the experiences of other caregivers would probably also be beneficial. Furthermore, a larger group of active members may possibly facilitate community sustainability [20,21]. Indeed, organizational commitment and financial and human resources are essential to maintain a community, and these efforts can be supported by the involvement of a larger group of people who provide care to aSAH patients [20]. As a result of the responses to the open questionnaire used in this pilot study, information will be added to the wiki section and rehabilitation specialists will become engaged in the MySAH community.

### Conclusions

In this pilot study, the online health community MySAH contributed to the aftercare of patients suffering from aSAH. There was easy access to information that was relevant for patients and families, which could be obtained from peers or caregivers. The MySAH community will, however, mainly be useful for a select group of patients because of differences in clinical outcomes.

## Multimedia Appendix 1

English translation of frequently asked questions in category “Can I…?” (In Dutch; Mag ik…?).

## Abbreviations

aSAH: aneurysmal subarachnoid hemorrhage
mRS: modified Rankin Scale
