# Supplementary material for: An Online Health Community for Aneurysmal Subarachnoid Hemorrhage Patients: A Pilot Study
Source: JMIR Res Protoc. 2014 Nov 13;3(4):e60. doi: 10.2196/resprot.3736 (PMC4260082; doi:10.2196/resprot.3736)
Supplement: Supplementary file 1 [file resprot_v3i4e60_app1.pdf]

Supplementary File 1:
English translation of frequently asked questions
in category "Can I…?" (In Dutch; Mag ik…?).

"Can I…?"

…. Fly? Yes You can!

After a subarachnoid hemorrhage, many people wonder if they are allowed to fly. They are afraid that the changed air pressure on the plane may cause a new bleed. Medically speaking, though, there is no reason not to fly.

…..Have sexual intercourse again?

Having sexual intercourse after a subarachnoid hemorrhage… No problem.

After a subarachnoid hemorrhage, many people wonder if they can have sexual intercourse again. They are anxious that a new bleed may occur. It is, however, safe to do so and there is no increased risk of a new hemorrhage.

It often happens that the physical effects of the hemorrhage (fatigue, headache) or feelings of anxiety or apathy affect your relationship and sex life. We recommend that you discuss this with your partner. It is also possible to speak about this with one of your healthcare professionals.

……Drive my car?

Driving my car after an aneurysm or subarachnoid hemorrhage: is it permitted?

The answer to this question depends on a number of factors. You can read more about government regulations in this blog.

If you have a disease or a condition in which fitness to drive is an issue, as a license holder you are obliged to report this to the Central Bureau for Driver Licences (Dutch: CBR) by way of a "self-declaration."

There are stricter regulations for people who use their license for professional purposes.

The conditions in which this applies are described in the 2000 Ministry of Transport and Water regulation: ‘Suitability Requirements.’ This regulation was last revised in 2008. Subarachnoid hemorrhage and cerebral aneurysms are both discussed in this regulation.

In summary, the following applies:
People with an accidentally-discovered aneurysm that has not bled, and is not being treated, are generally considered by the CBR to be appropriate to hold a driving license without any time limitations.
People with bleeding from an aneurysm should not drive until six months after the hemorrhage. After this period, when fitness to drive is not an issue, a person can be considered by the CBR to be suitable to hold a driving license. When there is doubt about a dysfunction that may bring the issue of safety into question, the individual’s driving must be assessed. In particular, a test drive with an expert from the CBR is required. The CBR has a comprehensive protocol for the test drive procedure. When the driving is assessed as being good, a license is approved for a term of three years.
If you have any questions about your situation and resuming driving, please discuss these with your healthcare professionals.

…… Drink alcohol?

A nice glass of wine? You can!

When you are treated for a ruptured aneurysm, you might wonder whether you can drink alcohol. There is no harm in doing so, as long as it’s in moderation. Keep in mind the medication that you are taking and always read the warnings in the leaflet that comes with the drugs.

….. Practice sport?

When you are diagnosed with a subarachnoid hemorrhage due to an aneurysm, you might wonder whether you can take part in sport. There is no harm in doing so. In fact, once you’re ready, it is good for your health to participate in sports again.
Please do be aware that you need to build up your physical endurance. However, we recommend gradually increasing the duration, intensity and frequency of your exercise.

…..Go to the sauna?

It is often asked whether a visit to the sauna is possible after a subarachnoid hemorrhage. The answer to this question is ‘Yes.’

A visit to the sauna can be relaxing and is therefore for good for you. Once there, like for everyone else, it is important that you drink enough. If you have high blood pressure, it is even more important that you adhere to the sauna regulations. In the sauna, your blood pressure goes down because the blood vessels expand. When cooling off, the opposite occurs. In people with high blood pressure, these fluctuations are greater, meaning that you should stick closely to the requirements of the establishment.
